# Different cervical vertebral bone quality scores for bone mineral density assessment for the patients with cervical degenerative disease undergoing ACCF/ACDF: computed tomography and magnetic resonance imaging-based study

**DOI:** 10.1186/s13018-023-04422-z

**Published:** 2023-12-06

**Authors:** Zhe Wang, Jingyao Zhang, Qian Chen, Yong Huang, Yueming Song, Limin Liu, Ganjun Feng

**Affiliations:** 1 Department of Orthopedics, Orthopedic Research Institute, West China Hospital, Sichuan University, Chengdu, China; 2 Core Facilities of West China Hospital, Sichuan University, Chengdu, China

**Keywords:** Cervical vertebrae, Osteoporosis, MRI, CT, Bone density

## Abstract

**Background:**

Bone mineral density (BMD) is important for the outcome of cervical spine surgery. As the gold standard of assessing BMD, dual-energy X-ray absorptiometry scans are often not ordered or go unreviewed in patients’ charts. As the supplement, MRI-based vertebral bone quality (VBQ) was found to accurately predict osteopenia/osteoporosis and postoperative complications in lumbar spine. However, discussion of the efficiency of VBQ in cervical spine is lacking. And measurement methods of VBQ in cervical spine are diverse and not universally acknowledged like lumbar spine. We aimed to compare the predictive performance of three kinds of different Cervical-VBQ (C-VBQ) scores for bone mineral density assessment in patients undergoing cervical spine surgery. HU value of cervical spine was set as a reference.

**Methods:**

Adult patients receiving cervical spine surgery for degenerative diseases were retrospectively included between Jan 2015 and Dec 2022 in our hospital. The VBQ scores and HU value were measured from preoperative MRI and CT. The correlation between HU value/C-VBQs (named C-VBQ1/2/3 according to different calculating methods) and DEXA T-score was analyzed using univariate linear correlation and Pearson’s correlation. We evaluated the predictive performance of those two parameters and achieved the most appropriate cutoff value by comparing the receiver operating characteristic (ROC) curves.

**Results:**

106 patients (34 patients with T ≥ − 1.0 vs 72 patients with T < − 1.0) were included (mean age: 51.95 ± 10.94, 48 men). According to Pearson correlation analysis, C-VBQ1/2/3 and HU value were all significantly correlated to DEXA T-score (Correlation Coefficient (*r*): C-VBQ1: − 0.393, C-VBQ2: − 0.368, C-VBQ3: − 0.395, HU value: 0.417, *p* < 0.001). The area under the ROC curve (AUC) was calculated (C-VBQ1: 0.717, C-VBQ2: 0.717, C-VBQ3: 0.727, HU value: 0.746). The AUC of the combination of C-VBQ3 and HU value was 0.786. At last, the most appropriate cutoff value was determined (C-VBQ1: 3.175, C-VBQ2: 3.005, C-VBQ3: 2.99, HU value: 299.85 HU).

**Conclusions:**

Different MRI-based C-VBQ scores could all be potential and alternative tools for opportunistically screening patients with osteopenia and osteoporosis before cervical spine surgery. Among them, C-VBQ calculated in ASI_C2–C7_/SI_T1-CSF_ performed better. We advised patients with C-VBQ higher than cutoff value to accept further BMD examination.

## Introduction

Cervical spine surgeries are challenging for patients with poor bone quality and complications such as cage subsidence, vertebral compression fractures, pseudoarthrosis, and instrumentation failure occur at significantly higher rates in osteoporotic patients due to difficulty in obtaining sufficient fixation [1–4]. The rate of osteoporosis in patients over 50 years old who underwent spine operations is 51.3% among females and 14.5% among males, which is higher than that of the general population [5].

However, according to osteoporosis guidelines, women aged > 65 years and men aged > 70 years are routinely recommended for dual-energy X-ray absorptiometry(DEXA) test, thus creating an age gap between osteoporosis generating (50 y) and advised DEXA test (65 y/70 y) [6]. For premenopausal women whose osteoporosis/osteopenia is challenging to diagnose [7], the prevalence is also not negligible according to Spanish research: 0.34% in the group aged 20–44 years; 4.31% in the group aged 45–49 years [8].

While DEXA is the gold standard for the diagnosis of osteopenia/osteoporosis [9], a low adherence rate for eligible patients to the guideline was found [10]. DEXA scans are often not ordered or go unreviewed in patients’ charts [11–13]. Clinically, a large number of patients receiving spine surgery do not have accessible BMD data. Thus, there is an urgency for a new predicting tool for preoperative BMD assessment to serve as a supplement to DEXA examination for optimizing spine surgery outcomes and mitigating the risk of complications [14].

Due to the aforementioned reasons, some researchers have sought to study novel alternative assessment measurements based on data acquired during routine preoperative evaluation. For example, computerized tomography (CT) has been introduced to predict osteopenia/osteoporosis and postoperative complications with good sensitivity and specificity in the cervical spine [15–17]. However, considering the radiation of CT which is 30 times higher than a spinal DEXA [18], recently, a novel alternative assessment measurement based on MRI called vertebral bone quality (VBQ) was described which has an 81% accuracy for lumbar spine [19–21].

The science behind the VBQ method is that fatty infiltration of trabecular bone which correlates negatively to bone density could provide potential information for assessing BMD. However, to date, whether the VBQ is applicable and how to calculate it in the cervical spine remains undetermined. Different conclusions and measurement ways such as ASI_C2–C7_/SI_T1-CSF_, MSI_C3–6_/SI_C5-CSF_, and MSI_C3–6_/SI_C2-CSF_ were reported [4, 21, 22].

As far as we know, hitherto, the predictive value of those two parameters (HU value and C-VBQ1/2/3) for preoperative BMD assessment in the cervical spine has never been directly compared together. The purpose of the present study is to compare them by selecting DEXA T-score as standard.

## Materials & methods

### Patient population

**Fig. 1:**
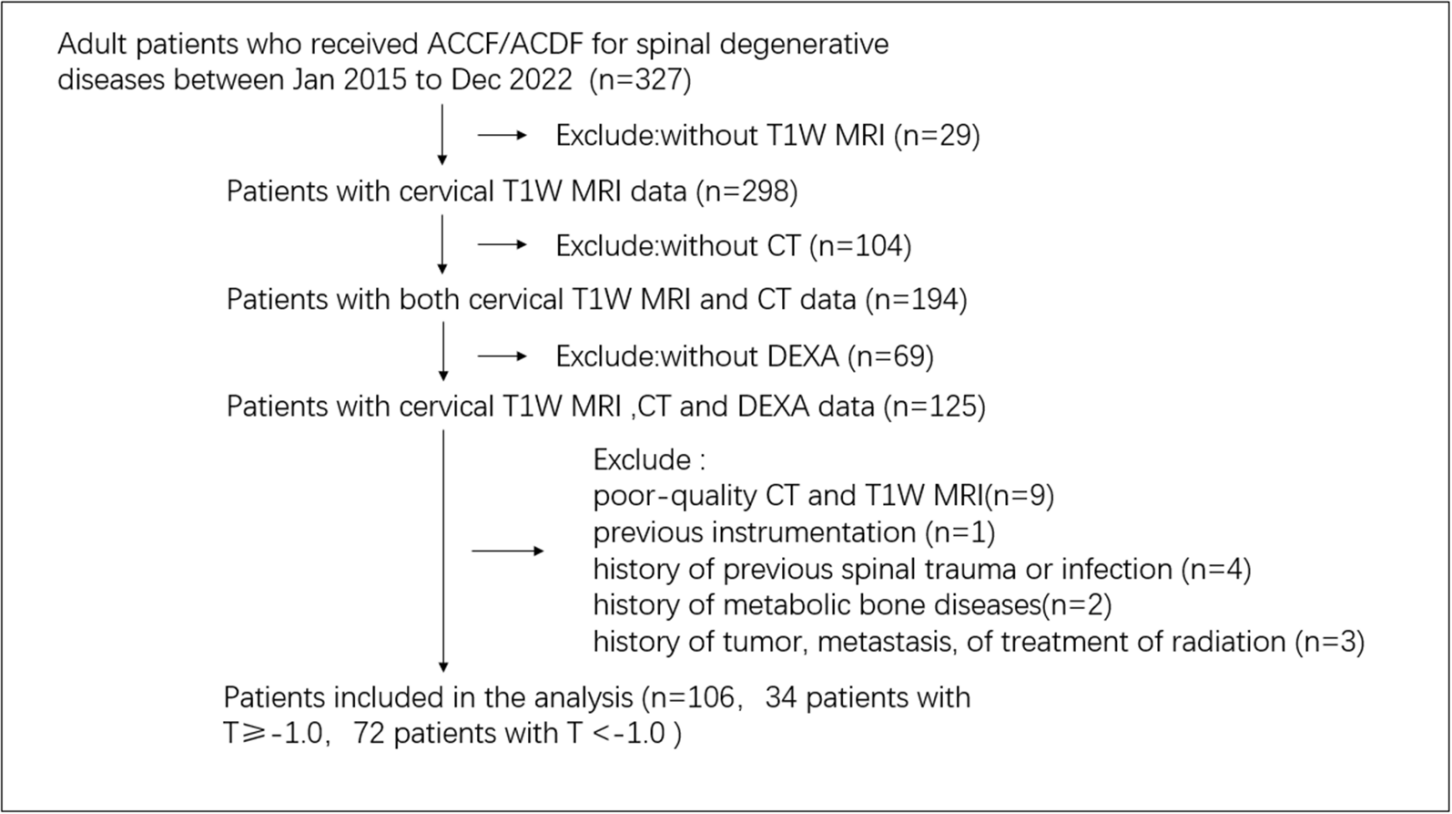
The flow diagram of the patient inclusion process

The study had institutional review board exemptions by our hospital. The requirement for informed consent from the participants was waived because of its retrospective nature. The study has been reported in line with the STROBE statement [23]. By consecutively retrieving the medical records of patients that underwent cervical spine surgery (including Anterior cervical corpectomy and fusion/Anterior cervical discectomy and fusion and so on) from Jan 2015 to Dec 2022, 106 patients with available preoperative DEXA (T-scores), CT, and T1-weighted MRI of the cervical spine that were no more than 12 months apart from each other were included in this study. Exclude criteria: age < 18; with previous cervical instrumentation; with poor-quality MRI and CT due to motion artifact; with a history of metabolic bone diseases other than osteopenia or osteoporosis; with evidence of tumor, metastasis, or treatment of radiation. Eligible patients were divided into 2 groups (normal group and osteopenia/osteoporosis group) according to DEXA T-score (T-score ≥ − 1.0 vs T-score < − 1.0). The sample selection process was shown in Fig. 1. Electronic medical records were retrospectively queried to collect demographic data including age, gender, body mass index (BMI), race, smoking status, history of alcohol abuse, long-term drug history of steroid use, and medical comorbidities. Siemens syngo imaging picture archiving and communication system (PACS) was utilized for all radiographic data collection (Siemens, Malvern, USA).

### Computed tomography measurements

**Fig. 2:**
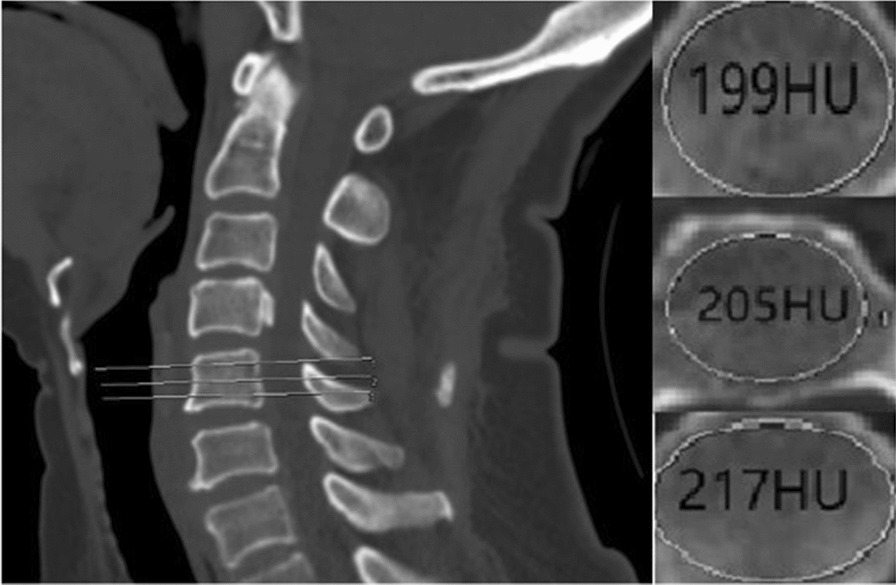
Illustration of the cervical CT HU Value calculation process. **a** Midsagittal slice of the vertebral body demonstrating three axial planes of interest (white transverse line). **b** Axial images showing region of interest (ROI) of HU values (white ellipse) generated by the imaging software program (inferior to the upper endplate; in the middle of the vertebral body; superior to the lower endplate corresponding to **a**

All subjects were scanned with a 64-slice multi-detector CT scanner (Siemens Somatom Definition AS, Malvern, USA) with a tube voltage of 120 kV. HU value measurements were obtained from Siemens Syngo Imaging PACS. According to the HU value’s calculation method raised by Schreiber et al., regions of interest were measured on the axial images at C2 through C6 at three separate locations: immediately inferior to the superior end plate, in the middle of the vertebral body, and superior to the inferior end plate[24] (Fig. 2). For each measurement, the largest possible elliptical region of interest (ROI) was drawn, excluding the cortical margins to prevent volume averaging. Measurements were performed by two independent observers (WZ and JYZ) and were then averaged.

### VBQ measurement and score calculation

**Fig. 3:**
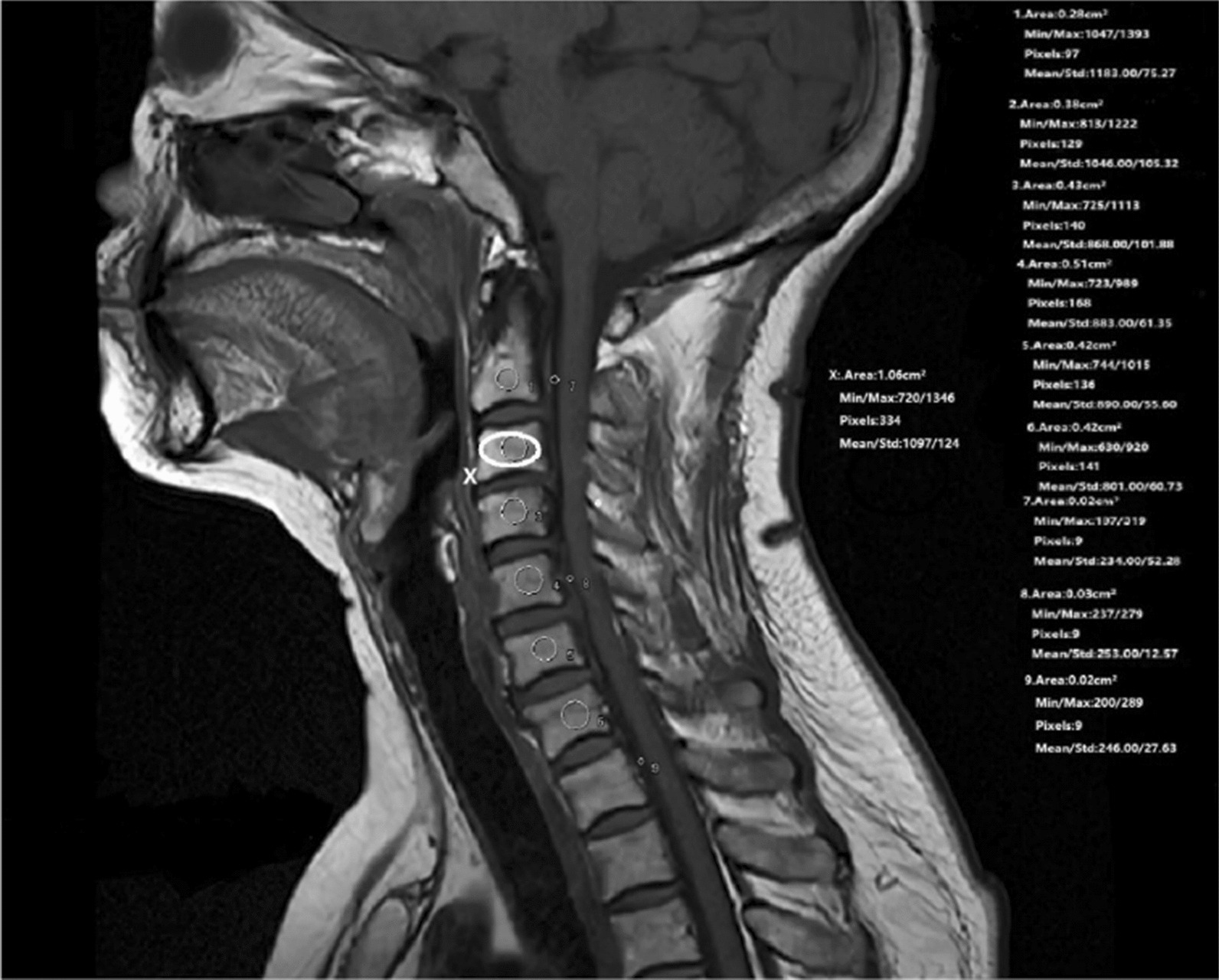
A Illustration of the cervical vertebral bone quality (C-VBQ) calculation process. ROI 1–6 was placed in medullary part of C2–T1 vertebrae, ROI 7, 8, 9 was placed in cerebrospinal fluid at C-2, C-5, T-1 level (Circles drawn with thin white line). Ellipse drawn with thick white line (noted *X*) was illustration of larger ROI of C-VBQ3

VBQ measurements were taken by placing ROI within the medullary portions of the vertebral bodies and within the cerebrospinal fluid on a midsagittal T1-weighted MRI image (avoid some structures such as the venous plexus), based on the lumbar spine measurement previously described by Ehresman et al. [20], as shown in Fig. 3. The elliptical ROI of C-VBQ3 is larger to maximize the inclusion of vertebral body cancellous bone; the circular ROI of C-VBQ1/2 was placed 3 mm from the perimeter of the vertebral body. For patients with abnormalities that prevented the vertebral ROI placement on the midsagittal slice, such as a focal lesion, venous plexus, or scoliotic change, a parasagittal cut was used. After that, C-VBQ scores obtained from different measurement methods were calculated by dividing the median/averaged signal intensity (SI) of the vertebral body by the mean signal intensity of the cerebrospinal fluid (SI-CSF) according to corresponding stipulation [4, 21, 22]. The difference in calculating methods is the selecting segment of cervical vertebrae, levels of cerebrospinal fluid and region of interest (ROI). C-VBQ1 was calculated using the quotient of median signal intensity (MSI) of the C3–C6 vertebrae divided by the mean signal intensity of C2 CSF. Similarly, C-VBQ2 was calculated using the quotient of median signal intensity (MSI) of the C3–C6 vertebrae divided by the mean signal intensity of C5 CSF. C-VBQ3 was calculated using the average signal intensity (ASI) of the C2–C7 vertebrae divided by the mean signal intensity of T2 CSF. C-VBQ1/2/3 refers to MSIC3-C6/SI C2-CSF, MSIC3-6/SIC5-CSF and ASIC2-7/SIT1-CSF respectively. All VBQ measurements were taken with the hospital’s picture archiving and communication system (PACS) software (Siemens syngo image, Malvern, USA). All VBQ measurements were taken by two trained research people (QC and HY) who were blinded to the status of the HU value. When the two sets of measurements differed by more than 10%, a third author (GJF) was consulted and the outlier value was removed.

### Statistical analysis

All data were recorded with Microsoft Excel (Microsoft, Redmond, WA, USA) and analyzed using SPSS Statistics version 27.0 (IBM Corp. Armonk, NY, USA). Continuous variables were described as mean ± standard deviation (SD) and categorical variables are expressed as a percentage/ratio. The normality of continuous variables was tested by the Shapiro–Wilk test. The independent samples *t*-test (or the Mann–Whitney *U* test) and Pearson’s *χ*^2^ test (or Fisher’s exact test) were used to compare differences between groups. The correlation between C-VBQ1/2/3 and DEXA T-score and the correlation between HU value and DEXA T-score was analyzed with Pearson correlation and univariate linear regression. Correlation coefficients were categorized as weak, moderate, and strong corresponding to value ranges of 0–0.3, 0.3–0.7, and 0.7–1, respectively [25]. Receiver operating characteristic (ROC) analysis was also performed to calculate the area under-curve (AUC) of the C-VBQ1/2/3, HU value, and the combination of those two measurements as a predictor of osteopenia/osteoporosis. In general, an AUC > 0.7 indicates a useful test [26]. The Youden Index was applied to obtain a satisfied cutoff value for diagnosis. The images of 15 randomly selected patients were used to measure C-VBQ and HU values in each method by two authors twice two months apart. The inter-class correlation coefficient (ICC) was used to calculate the inter-rater reliability and intra-rater reliability and was defined as follows: ICC less than 0.40 as poor, 0.40–0.59 as fair, 0.60–0.74 as good, and greater than 0.75 as excellent [27]. Statistical significance was set at *p* < 0.05.

## Results

### Patient population

As Fig. 1 shows, A total of 106 patients were included in the final analysis (mean age: 51.95 ± 10.94, 48 men). According to DEXA T-Score, 34 patients were categorized into the normal BMD group (T-Score ≥ − 1.0), and the other 72 patients were grouped into the osteopenia/osteoporosis group (T-Score < − 1.0). All demographics and radiological details of the study population are shown in Table 1. No significant differences in sex (*p* = 0.891), age (*p* = 0.790), BMI (*p* = 0.684), smoking status (*p* = 0.422), history of alcohol abuse (*p* = 0.666), history of steroid use (*p* = 1.000), or medical comorbidities (*p* = 0.835 for Anemia, *p* = 0.399 for Hyperlipidemia, *p* = 0.831 for Diabetes) were found between groups (Table 1). According to students’ *t*-tests, a significant difference of C-VBQ1/2/3 and HU value could be found between groups, which was consistent with the findings of the T-score. The interobserver reproducibility was excellent: C-VBQ1: ICC 0.891 (95% CI 0.881–0.902); C-VBQ2: ICC 0.875 (95% CI 0.860–0.889); C-VBQ3: ICC 0.900 (95% CI 0.886–0.913) and HU value: ICC 0.920 (95% CI 0.910–0.930). The ICC of intra-observer was also excellent: C-VBQ1: ICC 0.905 (95% CI 0.881–0.928); C-VBQ2: ICC 0.899 (95% CI 0.872–0.926); C-VBQ3: ICC 0.914 (95% CI 0.900–0.929) and HU value: ICC 0.925 (95% CI 0.902–0.949).

**Table 1:** Patient demographics

Factors	Normal (*n* = 34)	Osteopenia/osteoporosis (*n* = 72)	*p*
Mean age (SD)	51.47 ± 10.67	50.86 ± 11.12	0.790
Sex (male:female)	15:19	33:39	0.891
BMI (kg/m^2^)	24.67 ± 4.2	24.33 ± 3.95	0.684
Asian	34 (100%)	72 (100%)	1.000
Anemia (yes:no)	4:30	6:66	0.835
Hyperlipidemia (yes:no)	8:26	12:60	0.399
Diabetes (yes:no)	3:31	4:68	0.831
Alcoholism (yes:no)	9:25	22:50	0.666
Cigarette (yes:no)	10:24	16:56	0.422
Steroid use (yes:no)	2:32	5:67	1.000
CT(HU)	329.27 ± 59.79	269.44 ± 67.38	< 0.001*
VBQ-1	2.86 ± 0.64	3.36 ± 0.57	< 0.001*
VBQ-2	2.66 ± 0.56	3.12 ± 0.62	< 0.001*
VBQ-3	2.67 ± 0.63	3.18 ± 0.61	< 0.001*

*SD* Standard deviation; *BMI* Body mass index; *HU* Hounsfield Units; *VBQ* vertebral bone quality

*Statistical significance between two groups

### Correlation between the C-VBQs/HU value and DEXA T-score

**Fig. 4:**
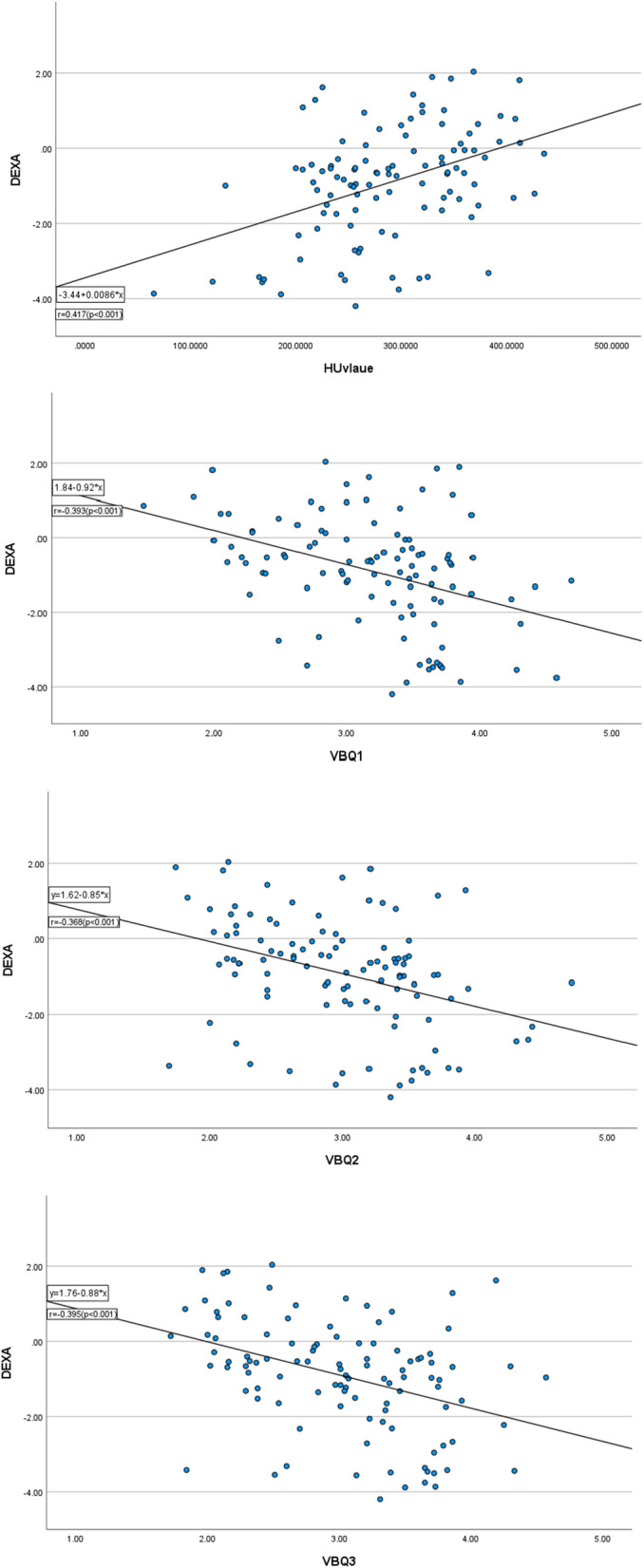
Linear regression and correlations between HU value/C-VBQ1/2/3 and DEXA T-Score

The correlation between three kinds of C-VBQs/HU value and DEXA T-score is shown in Table 2 and Fig. 4. Based on the data acquired in this study, the univariate linear regression showed that the higher C-VBQs score could independently indicate the presence of the lower DEXA T-Score with statistical significance. And the higher HU value could independently indicate the higher DEXA T-Score. As Table 2 indicated, C-VBQ1/2/3 and HU value were all found to be significantly correlated with the DEXA T-score according to Pearson correlation analysis. Among C-VBQ1/2/3, C-VBQ3 (ASI_C2–C7_/SI_T1-CSF_) showed a slightly better correlation coefficient (*r* = − 0.395, *p* < 0.001). HU value showed the highest correlation coefficient (*r* = 0.417, *p* < 0.001).

**Table 2:** Correlation between the Cervical VBQ Scores/HU value and DEXA T-Score

Linear regression formula (*Y*: T-score, *X*: C-VBQs/HU value)		Correlation-coefficient (*r*)	*p*
HU value	*Y* = 0.0086*X − 3.44	0.417	< 0.001*
VBQ-1	*Y* = − 0.92*X + 1.84	− 0.393	< 0.001*
VBQ-2	*Y* = − 0.85*X + 1.62	− 0.368	< 0.001*
VBQ-3	*Y* = − 0.88*X + 1.76	− 0.395	< 0.001*

*VBQ* vertebral bone quality; *DEXA *dual-energy X-ray absorptiometry; *HU* Hounsfield Units

*Statistical significance between two groups

### ROC analysis

**Fig. 5:**
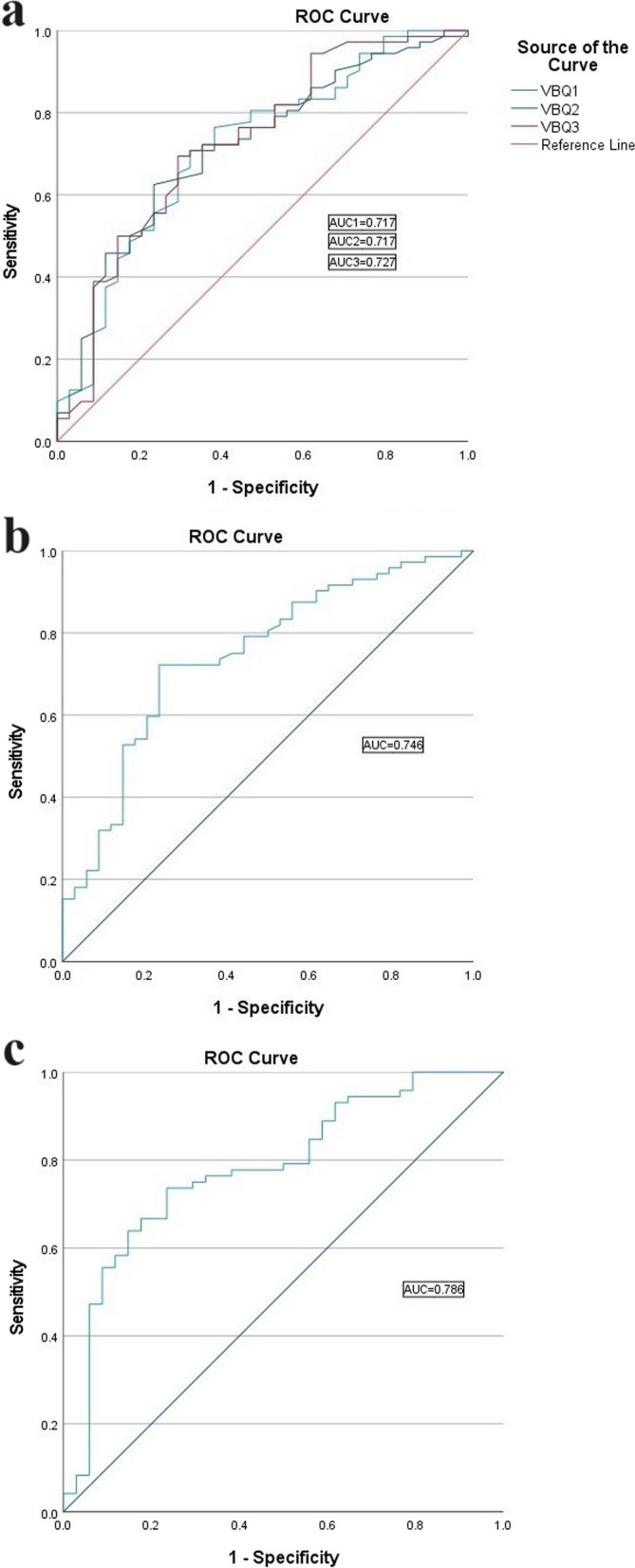
**a** ROC curve of the C-VBQ1/2/3. **b** ROC curve of the HU value. **c** ROC curve of the combination of C-VBQ3 and HU value

To evaluate the diagnostic efficiency, the ROC curve of C-VBQ1/2/3 and HU value was drawn and shown in Fig. 5a, b. According to the calculation based on the ROC curve, the most appropriate cutoff values were calculated (Table 3). At last, the combination of C-VBQ3 and HU value had the highest AUC of 0.786 (95% CI 0.686–0.886) (Fig. 5c).

**Table 3:** The area under the ROC curve (AUC) and a cutoff value of C-VBQ1/2/3 and HU value

	AUC (95% CI)	Cutoff value	Youden index	Sensitivity	Specificity
HU value	0.746 (0.645–0.846)	299.85 HU	0.487	0.722	0.765
C-VBQ1	0.717 (0.611–0.823)	3.175	0.385	0.708	0.676
C-VBQ2	0.717 (0.614–0.820)	3.005	0.390	0.625	0.765
C-VBQ3	0.727 (0.621–0.834)	2.990	0.400	0.694	0.706
VBQ3-HU	0.786 (0.686–0.886)				

*ROC* Received operating curve; *CI* Confidential interval

## Discussion

It is the first study to compare the predictive value of different MRI-based C-VBQs for preoperative BMD assessment in the cervical spine and set HU value as a reference. We found that different C-VBQs and HU value all had moderate and significant correlation with DEXA T-score. C-VBQ1/2/3 and HU value all had good AUC. Especially, C-VBQ3 in the measurement method of ASI_C2–C7_/SI_T1-CSF_ showed a slightly better correlation coefficient and AUC. Maybe the reason is that it was not that difficult to acquire the T1-level CSF to avoid the measuring difficulty, potential errors due to the presence of the intumescentia cervicalis and the possible spinal compression caused by degenerative tissues at C3–C7 in patients with degenerative diseases, which the other two C-VBQ scores couldn’t realize [21]. The inclusion of C2 (the second cervical vertebrae), larger ROI, and averaged signal intensity of cervical vertebrae could make the C-VBQ more representative and reflect more information about the cervical spine. It is worth mentioning that the other two types of C-VBQs were also accurate and acceptable to supply alternative BMD assessment. HU value had the highest correlation coefficient and AUC possibly because of the simplicity of the calculating process compared to C-VBQ and similar image-forming principle with DEXA based on X-ray. At last, the combination of C-VBQ3 and HU value had the highest AUC which meant the combination of those two predicting methods will be more accurate for screening patients. The interobserver reproducibility was excellent for both parameters. Thus, we advised people who have a preoperative HU value less than 300HU or C-VBQ3 more than 2.99 to accept further osteoporosis/osteopenia examination.

Low bone quality (osteopenia/osteoporosis) was found to be a risk factor leading to construct failure including screw pull-out and cage subsidence as the bony elastic modulus and strength decreased [28, 29]. Screening for low bone quality preoperatively may facilitate early interventions, such as antiresorptive medication (e.g.,, bisphosphonate, denosumab, raloxifene) and anabolic therapy (e.g.,, teriparatide), and can lead to alterations in the surgical plan [30]. However, as the gold standard of operative BMD assessment, DEXA has low examination rate. Meanwhile, DEXA is not an infallible method and can result in potentially erroneous measurements owing to diverse reasons like superimposition effects (bone spur, aortic calcification, and sclerotic change) [31–33]. Besides, the DEXA scan data was commonly based on the femoral or lumbar spinal images, which might be inconsistent with the regional bone quality of the cervical spine. Thus, researchers have been exploring additional tools with simplicity and convenience to directly assess bone mineral density in the cervical spine. According to Schreiber et al. and Lee et al., several studies have proved that the cervical spine’s HU value could be a good alternative assessment and accurately reflect the BMD degree as DEXA [15, 24, 34]. As for MRI-based VBQ which has been proven a good accuracy of 81% in the lumbar spine [19–21], however, whether the VBQ method could be applied in the cervical spine nowadays is still undetermined and measurement methods of C-VBQ are not universally acknowledged like lumbar spine (Lumbar spine VBQ: SI_L1–L4_/SI_L3-CSF_) [20]. For example, the study by Razzouk et al. [22] applied the calculating method of MSIC3-6/SIC5-CSF and concluded that C-VBQ scores are distinct from lumbar VBQ scores and do not provide adequate surrogate values of lumbar VBQ. The study by Cathleen et al. which applied the calculating method of MSIC3-6/SIC2-CSF found the newly developed C-VBQ score has a strong, positive correlation with the lumbar VBQ score [4]. And a study by Huang et al. concluded that the radiation-free and cost-effective method could be a potential tool for screening patients adopting calculating methods of ASIC2-7/SIT2-CSF [21]. Hitherto, there isn’t any study that compared different C-VBQs to figure out the efficiency and calculating method of it. Meanwhile, whether cervical HU value can be used for preoperative BMD assessment in the cervical spine is also lacking of discussion. Thus, in this study, we tried to compare those two parameters.

Before operating a cervical spine surgery, MRI and CT tests are routinely performed for detailed information about the compression situation of the cervical cord and nerve root. Therefore, a screening method based on MRI or CT will be cost-effective and provide more chances to opportunistically find out patients with osteopenia and osteoporosis before the traditional BMD assessment. In a recent study by Aggarwal et al. [3] it was reported that incorporating the evaluation of BMD into the routine CT assessment can potentially increase the annual screening for osteoporosis by 5% across the National Health Services (NHS). Besides, using ROI in CT and MRI has merits for measuring the bone quality because it can exclude the sclerotic bone, caused by degeneration.

Despite that the science behind the C-VBQs and HU value is robust and their efficacy has been verified in the lumbar spine, their application in the cervical spine still requires more surveys. As several other measurement methods of C-VBQ like MSIC2–T1/SI (cisterna magna) were newly proposed [35], more comparison is needed. There are some limitations to the current study. Firstly, this study has some potential bias due to its retrospective nature and relatively small sample size. Secondly, ethnically homogenous patients’ data were from one institution that mostly would receive ACCF/ACDF surgery because of degenerative cervical disease. That may cause selection bias and lack of reliability. More research including other surgical styles and the general population is needed. Thirdly, as DEXA was tested on the lumbar spine or hips which couldn’t directly provide site-specific evaluation of cervical vertebrae, more research based on quantitative computed tomography (QCT) tested on cervical vertebrae is needed. To promote the generality of HU value and C-VBQs in actual clinical situations, the comparison of those two parameters from different equipment and testing parameters is needed to evaluate the accuracy of the cutoff value in different hospitals. Lastly, to predict complications after cervical spine surgery, the direct correlation between C-VBQs/HU value and specific complications like cage subsidence, vertebral compression fractures, pseudoarthrosis, and instrumentation failure is needed to verify the efficiency of C-VBQs and HU value to predict prognosis to guide surgeons’ clinical arrangement.

## Conclusion

Different MRI-based C-VBQ scores could all be potential and alternative tools for opportunistically screening patients with osteopenia and osteoporosis before cervical spine surgery. And C-VBQ3 with the measurement method of ASI_C2–C7_/SI_T1-CSF_ performed better among different C-VBQs. We advised patients with C-VBQ higher than cutoff value to accept further BMD examination.

## Data Availability

Data will be available by contacting Ganjun Feng, the corresponding author, at the above address.

## Abbreviations

ACCF: Anterior cervical corpectomy and fusion
ACDF: Anterior cervical discectomy and fusion
ASI: Averaged signal intensity
AUC: Area under the curve
BMD: Bone mineral density
BMI: Body mass index
CSF: Cerebrospinal fluid
CT: Computed tomography
DEXA: Dual-energy X-ray absorptiometry
HU: Hounsfield units
ICC: Intraclass correlation coefficient
MRI: Magnetic resonance imaging
MSI: Median signal intensity
NHS: National Health Services
PACS: Picture archiving and communication system
QCT: Quantitative computed tomography
ROC: Receiver operating characteristic
ROI: Region of interest
VBQ: Vertebral bone quality
